# Suppression of endoplasmic reticulum stress-dependent autophagy enhances cynaropicrin-induced apoptosis via attenuation of the P62/Keap1/Nrf2 pathways in neuroblastoma

**DOI:** 10.3389/fphar.2022.977622

**Published:** 2022-09-16

**Authors:** Randong Yang, Shurong Ma, Ran Zhuo, Lingqi Xu, Siqi Jia, Pengcheng Yang, Ye Yao, Haibo Cao, Liya Ma, Jian Pan, Jian Wang

**Affiliations:** ^1^ Institute of Pediatric Research, Children’s Hospital of Soochow University, Suzhou, China; ^2^ Department of Pediatric Surgery, Children’s Hospital of Soochow University, Suzhou, China

**Keywords:** cynaropicrin, apoptosis, autophagy, p62/Keap1/Nrf2, neuroblastoma

## Abstract

Autophagy has dual roles in cancer, resulting in cellular adaptation to promote either cell survival or cell death. Modulating autophagy can enhance the cytotoxicity of many chemotherapeutic and targeted drugs and is increasingly considered to be a promising cancer treatment approach. Cynaropicrin (CYN) is a natural compound that was isolated from an edible plant (artichoke). Previous studies have shown that CYN exhibits antitumor effects in several cancer cell lines. However, it anticancer effects against neuroblastoma (NB) and the underlying mechanisms have not yet been investigated. More specifically, the regulation of autophagy in NB cells by CYN has never been reported before. In this study, we demonstrated that CYN induced apoptosis and protective autophagy. Further mechanistic studies suggested that ER stress-induced autophagy inhibited apoptosis by activating the p62/Keap1/Nrf2 pathways. Finally, *in vivo* data showed that CYN inhibited tumour growth in xenografted nude mice. Overall, our findings suggested that CYN may be a promising candidate for the treatment of NB, and the combination of pharmacological inhibitors of autophagy may hold novel therapeutic potential for the treatment of NB. Our paper will contribute to the rational utility and pharmacological studies of CYN in future anticancer research.

## Introduction

Neuroblastoma (NB), a malignant cancer originating from sympathetic nerves, is known to be the most common extracranial solid cancer in childhood, accounting for approximately 15% of all cancer-related paediatric deaths (Matthay et al., 2016; Youlden et al., 2020). Currently, there are multimodal strategies to treat NB, including surgery, radiotherapy, induction chemotherapy, immunotherapy and autologous stem cell transplantation either in combination or separately depending on the clinical features and disease stage (Cañete, 2020; Tas et al., 2020). However, the treatment of NB remains unsatisfactory, and the prognosis of advanced NB in children is poor, with a 5-year survival rate below 40% (Xu et al., 2018). Thus, there is a continuing need to develop new treatment strategies and drugs for NB.

Presently, there is mounting interest in the development of alternative medicines from plant-derived natural products with few side effects (Feng et al., 2011; Newman and Cragg, 2012; Fridlender et al., 2015). It has been estimated that roughly half of the drugs used in clinical practice have been derived from natural substances in recent years due to their anticancer activity (Kingston, 2011). Cynaropicrin (CYN), a sesquiterpene lactone, is a natural compound isolated from an edible plant (artichoke) (Zaib and Khan, 2020). Previous studies have reported that CYN exhibits phenomenal versatile biological and pharmacological abilities, including anti-inflammatory, antihepatitis C, antiparasitic, antibacterial, antiphotoaging, and antihyperlipidaemic properties and so on (Elsebai et al., 2016). More recently, the literature has proposed the antitumour efficacy of CYN, such as against colorectal cancer (Zheng et al., 2020), human melanoma (De Cicco et al., 2021), anaplastic thyroid cancer (Lepore et al., 2019), and lung carcinoma (Ding et al., 2021). However, to date, there are no studies on CYN with respect to NB, and the possible effects and underlying mechanisms of CYN on NB are still elusive.

It is currently well established from research that natural compounds possessing anticancer properties can induce apoptosis by modulating various intracellular pathways, including endoplasmic reticulum (ER) stress and autophagy (Elmore 2007; Nikoletopoulou et al., 2013; Hasima and Ozpolat, 2014). Notably, the functional association between apoptosis, ER stress and autophagy is an extensive crosstalk network (Song et al., 2017). The ER is an organelle that is responsible for protein folding and calcium homeostasis maintenance. ER stress occurs when homeostatic processes are disrupted and primarily results in the activation of the unfolded protein response (UPR). There are three distinct pathways in the UPR: ATF6, PERK-eIF2α-ATF4 and IRE1 (Healy et al., 2009). Furthermore, accumulating evidence has demonstrated the vital role of ER stress in apoptosis and autophagy in various tumour cells. Therefore, targeting the ER stress response is an effective anticancer strategy. To date, recent studies have not revealed the relationship between CYN and ER stress.

Autophagy, commonly referred to as macroautophagy, can be triggered by ER stress (Radogna et al., 2015). The autophagic pathway includes vesicle elongation, autophagosome formation, maturation, autophagosome–lysosome fusion, and degradation (Choi et al., 2013). Autophagy acts as a double-edged sword that facilitates or inhibits apoptosis induction in cancer cells under stress conditions (Su et al., 2015; Song et al., 2017). Increasing evidence has indicated that inhibiting autophagy induced by chemotherapy or targeted therapeutic drugs can lead to favourable conditions in anticancer therapy by promoting cancer cell apoptosis (Rangwala et al., 2014; Datta et al., 2019). Similarly, nuclear factor-erythroid 2-related factor 2 (Nrf2) is another cellular protective signalling pathway conferring adaptive protection against proteotoxic stress. Under physiological conditions, Nrf2 is carried to the proteasome by Keap1 and is maintained at a low level through degradation by the ubiquitin proteasome system (Schmoll et al., 2017). Under stress conditions, Nrf2 is released from the Keap1-Nrf2 complex and translocates into the nucleus (Itoh et al., 2003). In addition to this classical pathway, in the p62-dependent noncanonical Keap1-Nrf2 pathway, p62 persistently activates Nrf2 through competitive interaction with Keap1 (Celesia et al., 2020; Dash et al., 2020). Evidence has shown that the Nrf2 signal pathway is related to the regulation of autophagy in cells under ER stress. Therefore, in this study, we investigated the anticancer efficacy of CYN against NB and further elucidated the role and potential mechanisms of CYN on ER stress, autophagy and the Nrf2 pathway in NB cells.

## Materials and methods

### Reagents and antibodies

CYN (97% purity) was purchased from Chengdu Biopurify Phytochemicals Ltd. (Chengdu, China), and a stock solution (100 mM) in dimethyl sulfoxide (DMSO; ≥99.7%, Sigma‒Aldrich, St. Louis, MO, United States) was stored at −20°C. The final concentrations of DMSO in all experiments were lower than 0.1% (V/V). RAPA (100 nM), 3-Methyladenine (3-MA, 2 mM), chloroquine (CQ, 10 μM) and TUDCA (2 μM) were purchased from MCE (Monmouth Junction, NJ, United States). Triton X-100, 4% paraformaldehyde and a bicinchoninic acid (BCA) protein assay kit were purchased from Beyotime Biotechnology (Shanghai, China). Primary antibodies against Bax, Bcl-2, Beclin-1, LC3B, p62/SQSTM1, Atg5, CHOP, GRP78, ATF6, p-eIF2α, IRE1α and LAMP-2 were purchased from Abcam (Cambridge, United Kingdom). Primary antibodies against PARP, cleaved caspase-3, caspase-3, Ki-67 and GAPDH were purchased from Cell Signaling Technology (Danvers, MA, United States).

### Cell culture

The human NB cell lines (SK-N-BE(2) and SH-SY5Y) and human 293FT cells were obtained from the National Collection of Authenticated Cell Cultures (Shanghai, China). Cells were cultured in DMEM/F12 supplemented with 10% FBS (all from Gibco-BRL, Grand Island, NY, United States) and 1% penicillin‒streptomycin (Beyotime Biotechnology, Shanghai, China) at 37°C in a humidified atmosphere containing 5% CO_2_.

### Cell viability assay

SK-N-BE(2) and SH-SY5Y cells were seeded into 96-well plates (2 × 10^4^ cells per well) and incubated overnight. Then, the cells were treated with different concentrations of CYN or DMSO. After stimulation, cell proliferation was determined using CCK-8 reagent (Dojindo Laboratories, Kumamoto, Japan) every 24 h for four consecutive days. The absorbance at 450 nm was measured with a microplate reader (Thermo Fisher Scientific, Grand Island, NY). The viability of control cells was taken as 100%.

### EdU experiment

An EdU kit (Beyotime, Shanghai, China) was used for the EdU staining assay. According to the manufacturer’s instructions, the reaction solution was added to each well for 2 h of incubation in the dark. Afterwards, the cells were fixed with 4% paraformaldehyde for 30 min, and 0.3% Triton X-100 was added for 15 min at room temperature. After staining the nuclei with Hoechst 33342, the cells were collected under a fluorescence microscope (Olympus, Tokyo, Japan).

### Colony formation assay

NB cells were inoculated in six-well plates (1000 cells/well) and treated with different concentrations (0, 5, and 10 μM) of CYN. After culturing for 14 days, cell colonies were immobilized with 4% paraformaldehyde for 30 min and stained with 0.5% crystal violet (Boster, Wuhan, Boster) for 15 min.

### Lentivirus preparation and infection

For the downregulation of CHOP and Nrf2, shRNA sequences were synthesized by IGE Biotechnology (Guangzhou, China). For lentivirus preparation, the packaging plasmid psPAX2 and envelope plasmid pMD2.G were purchased from IGE Biotechnology (Guangzhou, China). 293FT cells were cotransfected with pMD2.G, psPAX2 and CHOP or Nrf2 plasmids for 6 h, and then the cell medium was removed for cell culture in fresh medium for 48 h. The viral supernatant was collected, filtered, and concentrated by PEG-8000 (Beyotime, Shanghai, China) precipitation. After incubation of the lentivirus with NB cells for 24 h, puromycin (Sigma‒Aldrich, St. Louis, MO, United States) was used to screen for stable cell lines.

### Cell cycle analysis

Flow cytometry was used to perform cell cycle analysis. NB cells were trypsinized after CYN treatment for 24 h, fixed with 75% ethanol at 4°C overnight, and then incubated with 50 μg/ml PI for 30 min at room temperature in the dark. Subsequently, the cells stained with PI fluorescence were detected with flow cytometry.

### Annexin V/PI staining

The extent of apoptosis was determined using an Annexin V/PI detection apoptosis kit (BD Biosciences, Heidelberg, Germany). After CYN treatment for 24 h, NB cells were trypsinized with trypsin solution without EDTA, harvested, washed with ice-cold PBS, and then resuspended in binding buffer. Subsequently, the cells were incubated with Annexin V and PI for 15 min at room temperature. After incubation, the results were assessed by flow cytometry (BD Biosciences, Heidelberg, Germany).

### Western blotting analysis

Total protein was lysed in RIPA buffer (Beyotime Biotechnology, Shanghai, China), and the cytoplasmic and nuclear proteins were extracted with a nuclear and cytoplasmic protein extraction kit (Beyotime Biotechnology, Shanghai, China). The protein samples were separated *via* SDS‒PAGE and transferred to PVDF membranes. The membranes were blocked with TBST containing 5% skim milk for 1.5 h and subsequently incubated with primary antibodies at 4°C overnight, followed by incubation with secondary antibodies at room temperature for 1 h. Finally, images of the western blot bands were analysed using an ECL system (Perkin Elmer, Waltham, MA, United States).

### Immunofluorescence assay

After the designated treatments, NB cells were fixed in 4% paraformaldehyde for 30 min, permeabilized with 0.3% Triton X-100 for 15 min, incubated in 5% BSA for 1 h (all the above steps were conducted at room temperature), and incubated with primary antibodies overnight at 4°C. The next day, Alexa Fluor 594 goat anti-rabbit IgG (Jackson, West Grove, PA, United States) or Alexa Fluor 488 goat anti-mouse IgG (Jackson, West Grove, PA, United States) was used as the secondary antibody. After incubation with the secondary antibody at room temperature for 1 h in the dark, the nuclei were stained with DAPI (Biosharp, Shanghai, China) for 5 min. Finally, the images were photographed by fluorescence microscopy (Olympus, Tokyo, Japan).

### Analysis of autophagy flux

NB cells were transfected with the mRFP-GFP-LC3 adenovirus (IGE Biotechnology, Guangzhou, China) for subsequent experimental studies for 24 h in DMEM/F12 supplemented with 10% FBS and 1% p/s. After washing with PBS, the cells were immediately fixed with 4% paraformaldehyde for 30 min, and 0.3% Triton X-100 was added for 15 min at room temperature. Subsequently, the nuclei were stained with DAPI for 5 min at room temperature. Autophagic flux measurements were performed with laser scanning confocal microscopes (Olympus, Tokyo, Japan).

### Coimmunoprecipitation assay

After treatment with CYN, the total cellular proteins were harvested by centrifugation and incubated with p62 or Keap1 antibody at 4°C overnight. Next, the cell lysates were incubated with protein A/G agarose beads for 4 h at 4°C. After elution from the bead-bound immunocomplexes, western blotting, as described for the western blot assay, was carried out to determine the protein levels.

### Transmission electron microscopy

Transmission electron microscopy (TEM) was used to visualize the autophagosomes and autolysosomes. NB cells were harvested by trypsinization, fixed with 2.5% glutaraldehyde overnight at 4°C, and postfixed with 1% OsO4 (pH 7.4) for 2 h at room temperature. After OsO4 removal, the samples were dehydrated in a graded ethanol series (30–100%), infiltrated, and embedded in resin. After polymerization of the resin at 55°C for 36 h, serial sections were cut with a Leica EM UC7 ultramicrotome (Leica, Nussolch, Germany). Sections were stained with uranyl acetate and alkaline lead citrate and then examined under a Gatan SC1000 (Model 832) CCD camera (Gatan, Pleasanton, CA, United States).

### *In Vivo* experiments

Female BALB/c nude mice (4 weeks old) received a subcutaneous injection of NB cells (2 × 10^6^ cells per mouse) suspended in 100 μl of extracellular matrix gel (Corning, NY, United States) into their front flanks. The mice were randomly divided into two groups (*n* = 5) and treated with CYN once a day for 3 weeks (0 or 5 mg/kg, i.g.). This concentration was referred to the article by Zhang et al. (Zheng et al., 2020). Tumour volumes and mouse weights were measured every 3 days. Tumour volumes were calculated with the formula (length × width × height)/2. After 3 weeks, all the mice were sacrificed by euthanasia, and afterwards, the tumours were quickly collected for weight measurements and then embedded in paraffin for immunohistochemistry.

### Immunohistochemistry

Tumour tissues were fixed in 4% paraformaldehyde and embedded in paraffin for sectioning. The sections were dewaxed in xylene and rehydrated in graded alcohol solutions. Primary antibodies (anti-Ki-67, anti-cleaved caspase-3, anti-Beclin-1, and anti-CHOP) were added for overnight incubation at 4°C, followed by staining with a secondary antibody (Thermo Fisher Scientific, Grand Island, NY) for 30 min at 37°C. An ultrasensitive SP (Mouse/Rabbit) IHC Kit and DAB Plus Kit (MXB Biotechnologies, China) were used following the manufacturer’s protocols. Before dehydration and mounting, the sections were counterstained with haematoxylin. Finally, images were examined using an Olympus microscope camera (Tokyo, Japan).

### Statistical analysis of data

All experiments were repeated at least three times, and the data are expressed as the mean ± SD. Statistical significance was analysed using *t* test (two groups) or one-way ANOVA (multiple groups), and a *p* value <0.05 was considered statistically significant.

## Results

### CYN inhibited the proliferation of NB cells

**FIGURE 1:**
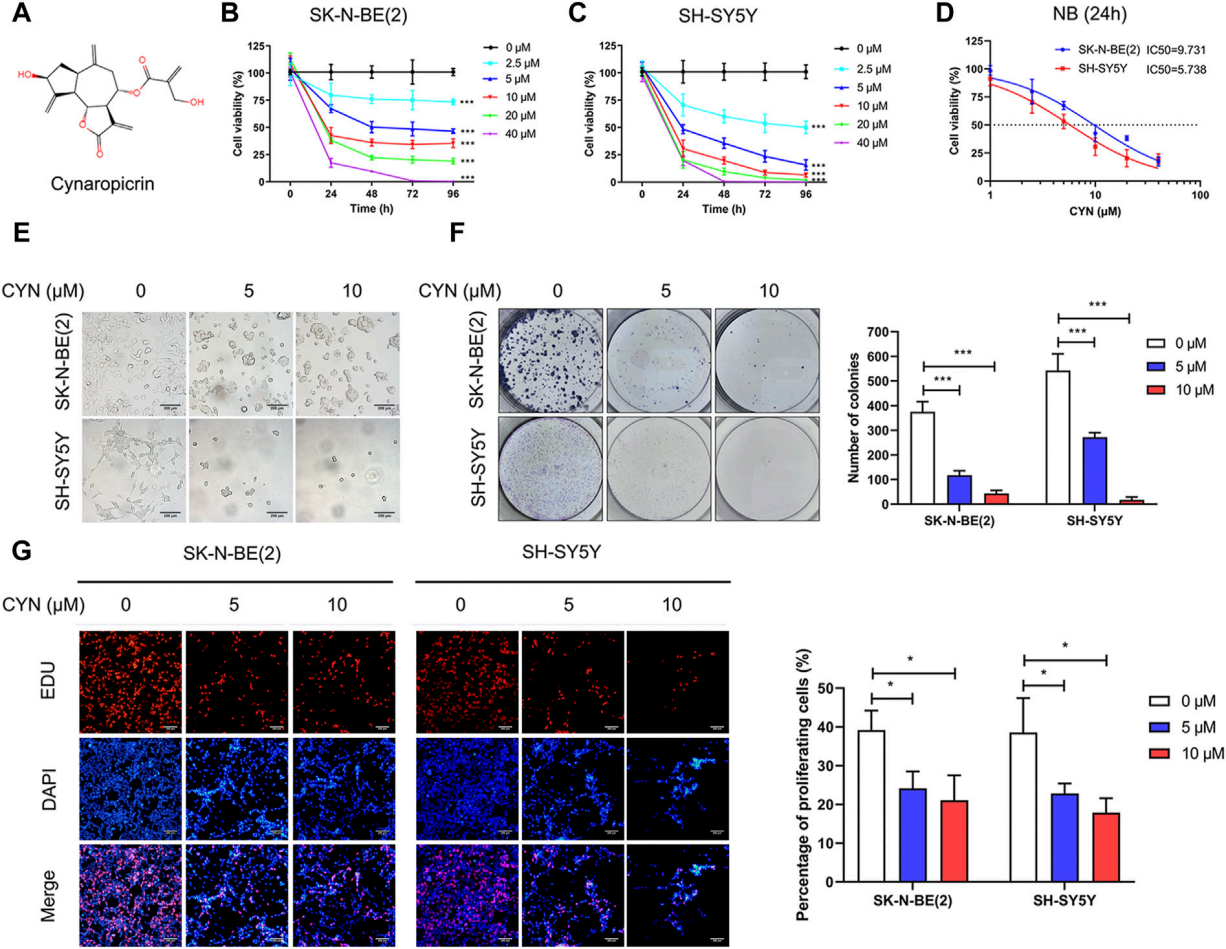
Effects of CYN on cell viability in NB cells. **(A)** The structure of cynaropicrin. **(B**,**C)** Cell viability was evaluated by CCK-8 assay. **(D)** IC50 was analyzed *via* the cell viability assay. **(E)** Morphological changes of NB cells (Scale bar = 200 µm). **(F)** Colony formation assay of NB cells treated with CYN. **(G)** EDU incorporation assay of NB cells treated with CYN (Scale bar = 200 µm). *p < 0.05, ***p < 0.001 versus the control group.

The chemical structure of CYN is shown in Figure 1A. To determine the effect of CYN on the proliferation of NB cells, the viability of NB cells treated with different concentrations of CYN (0, 2.5, 5, 10, 20 and 40 μM) for 24, 48, 72 and 96 h was investigated by CCK-8 assay. The results showed that CYN inhibited NB cell proliferation in a time- and dose-dependent manner (Figures 1B,C). Further analysis showed that the IC50 values for SK-N-BE(2) and SH-SY5Y cells were 9.731 and 5.738 μM at 24 h, respectively (Figure 1D). Therefore, concentrations of 5 and 10 μM were selected for the following experiments. Compared with the control, an increase in the number of floating dead cells and cell shrinkage, which are suggestive of cell death, were observed 24 h after CYN treatment (Figure 1E). Subsequently, a colony formation assay was performed to further examine the CYN-mediated inhibition of NB cell proliferation, and the results showed that CYN significantly inhibited colony formation (Figure 1F). Next, an EdU assay was used to examine the antiproliferative activities of CYN against NB cells. As shown in Figure 1G, CYN significantly suppressed DNA replication in NB cells. Collectively, these data proved that CYN had a significant inhibitory effect on the proliferation of NB cells.

### CYN induced cell cycle arrest and NB cell apoptosis

**FIGURE 2:**
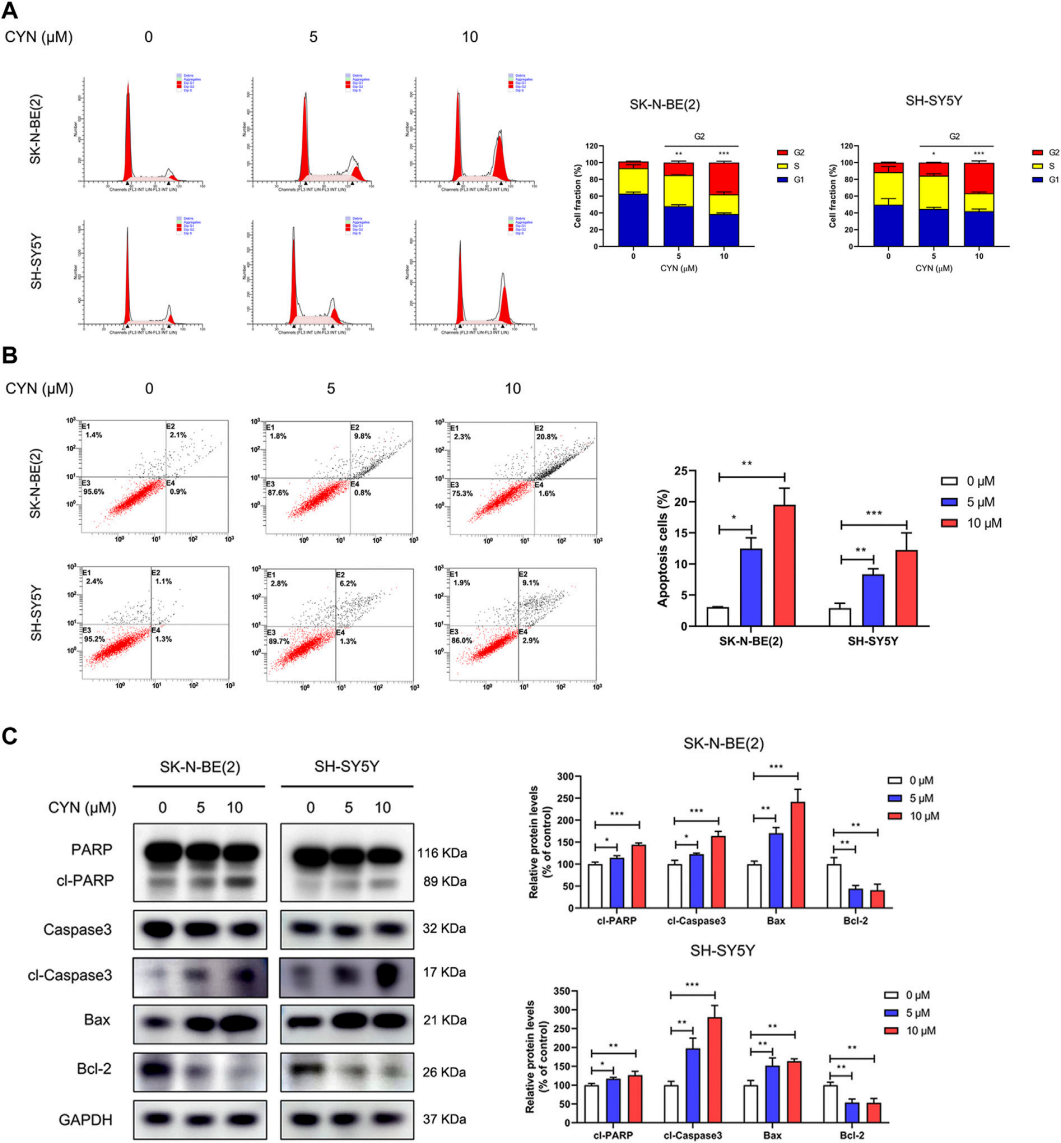
Effects of CYN on the cell cycle and apoptosis in NB cells. **(A)** Cell cycle was analyzed by flow cytometry. **(B)** Annexin V-FITC/PI staining and flow cytometry were used to measure and analyze apoptotic NB cells treated with CYN for 24 h. **(C)** The expression levels of apoptosis markers were measured by western blot analysis. *p < 0.05, **p < 0.01, ***p < 0.001 versus the control group.

To further determine whether cell cycle distribution and apoptosis participate in the inhibition of NB cell proliferation, we first investigated the effect of CYN on cell cycle distribution by flow cytometry. The results showed that CYN treatment markedly arrested NB cells at the G2 phase (Figure 2A). We next explored the effect of CYN on the apoptosis of NB cells using Annexin V/PI staining and flow cytometry. The flow cytometry data revealed that CYN significantly increased the apoptosis rates in NB cells with increasing dosage (Figure 2B). In addition, western blot analysis showed that the expression levels of cleaved PARP, cleaved caspase-3, and Bax were considerably upregulated, while the expression level of Bcl-2 was downregulated after treatment with CYN (Figure 2C). Thus, the above evidence illustrated that CYN affected the proliferation of NB cells by inducing cell cycle arrest and apoptosis.

### CYN initiated autophagy and inhibited autophagic flux in NB cells

**FIGURE 3:**
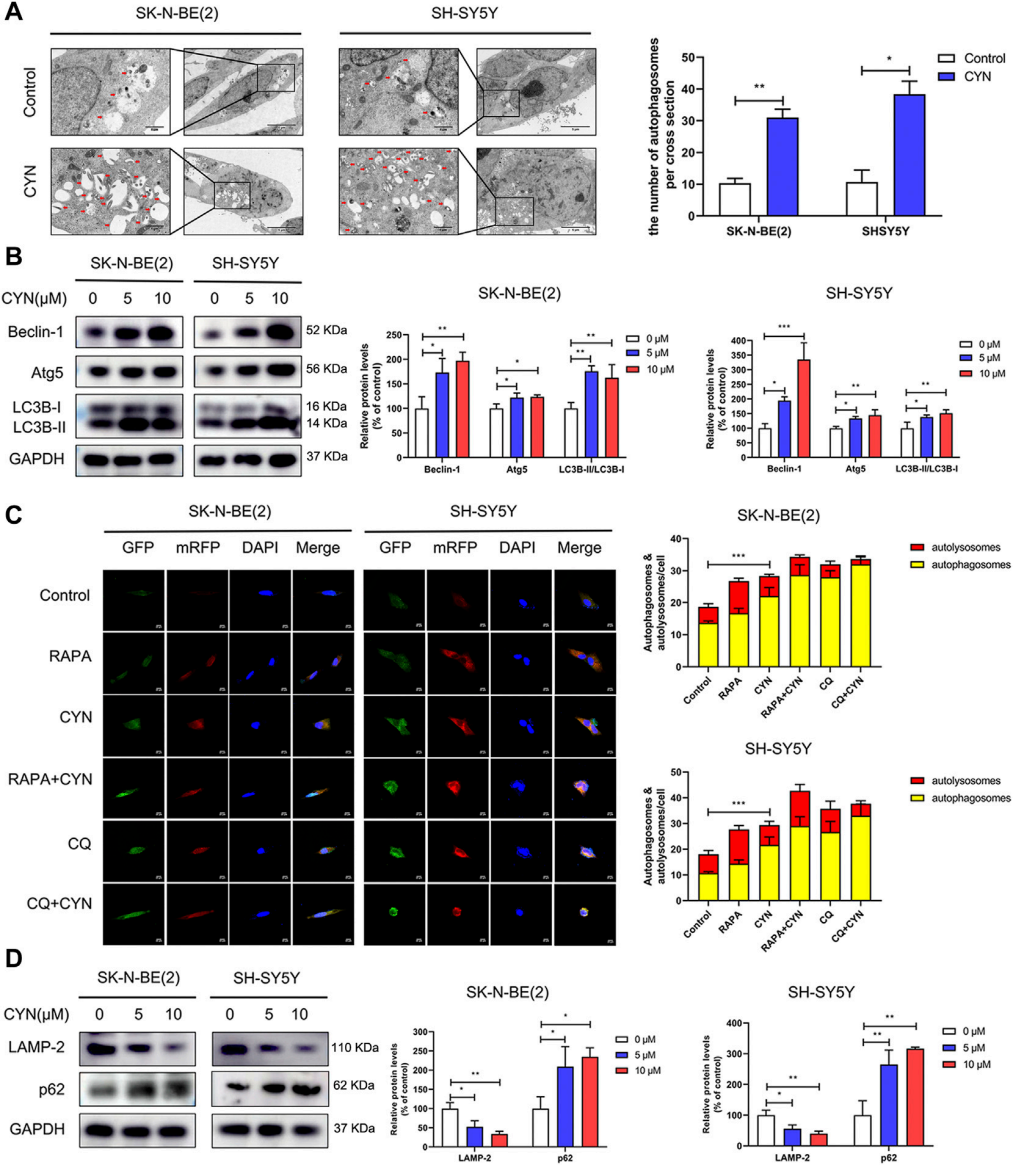
Effects of CYN on the autophagy level in NB cells. **(A)** Transmission electron microscopy (TEM) was utilized to observe the ultrastructure of NB cells, and the arrows indicate autophagosomes. **(B)** The expression levels of autophagy markers were analyzed by western blotting. **(C)** Confocal microscopy was utilized to detect fluorescence of mRFP-GFP-LC3. **(D)** Western blotting analysis of LAMP-2 and p62 protein expression. *p < 0.05, **p < 0.01, ***p < 0.001 versus the control group.

The correlation between autophagy and apoptosis in tumour cells has been previously reported (Rangwala et al., 2014; Su et al., 2015; Song et al., 2017; Datta et al., 2019). To assess CYN-induced autophagy, transmission electron microscopy (TEM) was used. The TEM images showed a notably increased number of autophagic vacuoles (Figure 3A, red arrow) in the cytoplasmic area of CYN-treated NB cells compared with the control group. We also evaluated the expression levels of autophagy-related proteins by western blotting. As shown in Figure 3B, CYN increased Beclin-1, Atg5 and LC3B-II protein levels and the LC3B-II/LC3B-I ratio.

Furthermore, autophagic flux, as a dynamic process, manifests as autophagosome formation and autophagic degradation. Blocking the fusion of autophagosomes and lysosomes impairs autophagic degradation (Mizushima et al., 2010). Thus, NB cells transfected with mRFP-GFP-LC3 adenovirus were applied to distinguish autophagosomes and autolysosomes. The GFP-tag in the mRFP-GFP-LC3 fusion protein is quenched, while the mRFP-tag remains in the acidic pH environment of autolysosomes. Therefore, in early autophagosomes, the red puncta overlaid with the green puncta and appeared yellow in the merged images, while in the autolysosomes, the free red puncta did not cover the green puncta and appeared red in the merged images. As shown in Figure 3C, RAPA (100 nM, an autophagy inducer) enhanced the formation of both green and red puncta, and colocalization gave rise to more yellow and red-only puncta, suggesting the accumulation of autophagosomes and the maturation of autolysosomes. Conversely, both CYN and CQ (10 μM, an inhibitor of autophagosome-lysosome fusion) dramatically increased the number of red and green puncta. After colocalization, yellow fluorescence significantly increased instead of red fluorescence, indicating that CYN autophagic flux blocked the autophagosome–autolysosome fusion process, similar to CQ. Next, we examined the fluorescence colocalization of LC3B and LAMP-2 LAMP-2, as an autophagosome-lysosomal fusion marker, can reflect the maturation of autolysosomes. Minimal colocalization of LC3B and LAMP-2 was found after CYN treatment (Supplementary Figure S1), which further confirmed that CYN can block autophagosome - lysosome fusion. Additionally, stimulation of autophagic flux can cause the depletion of p62, an autophagy adapter protein. The implication is that upon the impairment of autophagic degradation, p62 accumulates within cells. Herein, we found that CYN suppressed the expression of LAMP-2 and increased the expression of p62 in a dose-dependent manner (Figure 3D). Taken together, these results suggested that CYN-induced autophagosome accumulation was due to both the initiation of autophagy and the inhibition of autophagic flux.

### Autophagy stimulated by CYN partially attenuated apoptotic cell death in NB cells

**FIGURE 4:**
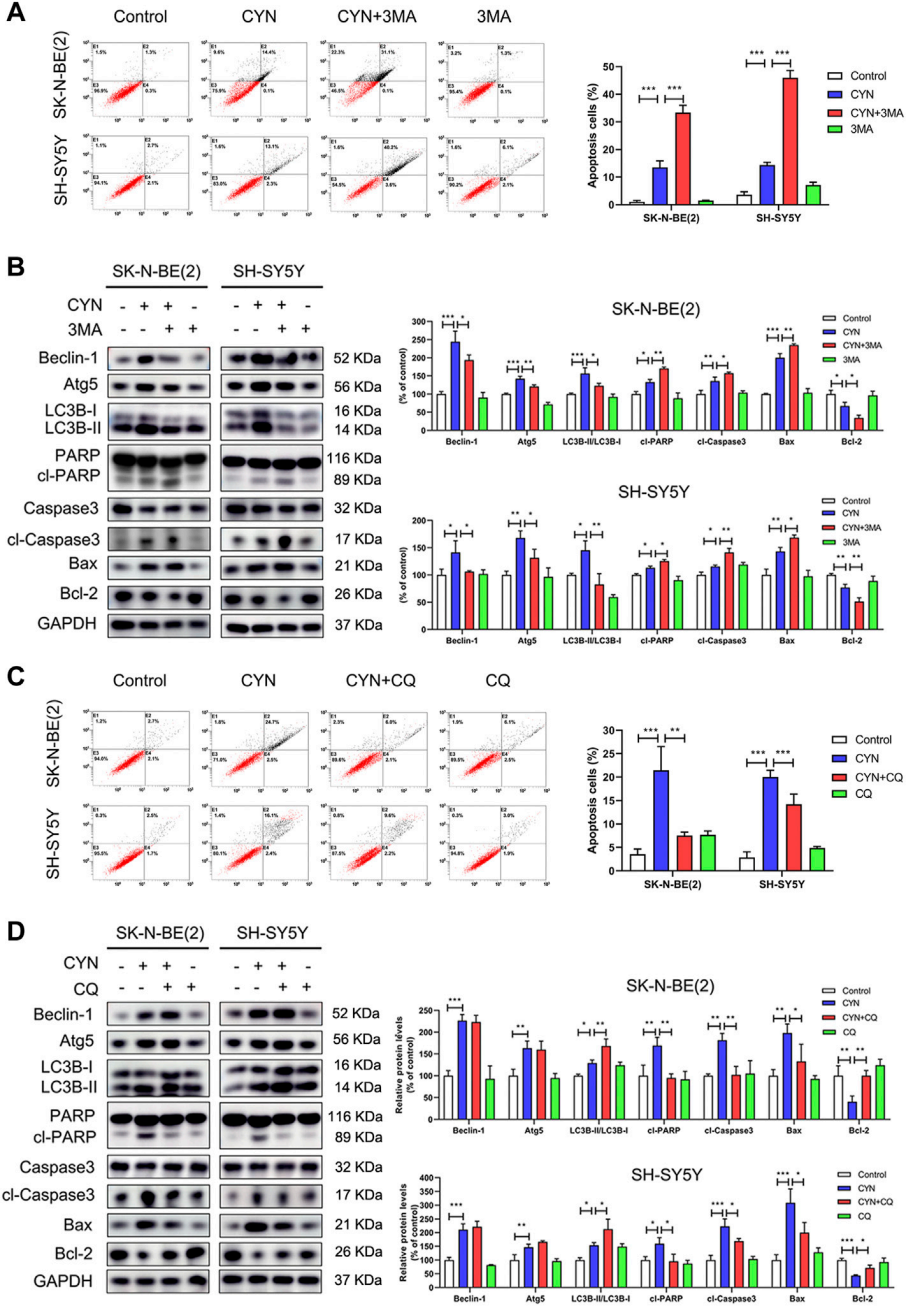
Relationship between apoptosis and autophagy in NB cells treated by CYN. **(A)** Apoptosis of NB cells was measured and analyzed by flow cytometry. **(B)** The expression levels of autophagy and apoptosis markers were analyzed by western blotting. **(C)** The number of apoptotic cells was assessed by Annexin V-FITC/PI staining and flow cytometry. **(D)** The expression levels of apoptosis markers were measured by western blot analysis. *p < 0.05, **p < 0.01, ***p < 0.001 versus the control group. *p < 0.05, **p < 0.01, ***p < 0.001 versus the CYN group.

Having clearly established that CYN activated autophagy in NB cells, we then attempted to determine the functional relationship between autophagy and apoptosis after CYN treatment. In our studies, the protein level of Atg5 was significantly increased in NB cells after CYN treatment (Figure 3B). To validate the relationship between CYN-induced autophagy and apoptosis, we silenced Atg5 by siRNA and used Annexin V-FITC/PI staining by flow cytometry and western blotting to examin apoptosis. The results showed that silencing of Atg5 significantly attenuated LC3-II accumulation and increased apoptosis in NB cells (Supplementary Figure S2). Additionally, Either 3 MA (2 mM, a specific early-phase autophagy inhibitor) or CQ (10 μM, a specific late-phase autophagy inhibitor) was used in NB cells. As illustrated in Figure 4A, cotreatment with CYN and 3-MA resulted in markedly more apoptosis than treatment with CYN (10 μM) alone. In addition, the western blot data showed that CYN and 3 MA cotreatment attenuated the effects of CYN on the expression levels of autophagy-related proteins (Beclin-1, Atg5 and LC3B) and increased the expression of apoptotic markers compared with CYN treatment (Figure 4B). The above results suggested that inhibiting autophagy with 3 MA enhanced the apoptotic effects of CYN in NB cells. Contrary to the above results, CQ partly abolished the induction of cell apoptosis induced by CYN (Figure 4C). Consistent with the results from flow cytometry, CQ effectively inhibited the CYN-induced upregulation of the expression of apoptotic markers (Figure 4D). In addition, CQ enhanced the effect of CYN on the expression level of LC3B but had no effect on Beclin-1 and Atg5. These data suggested that CYN played a protective role in CYN-induced NB cell apoptosis by initiating autophagy and blocking autophagosome-lysosomal fusion.

### NB cell apoptosis induced by CYN could Be partially abrogated by autophagy mediated by triggering ER stress

**FIGURE 5:**
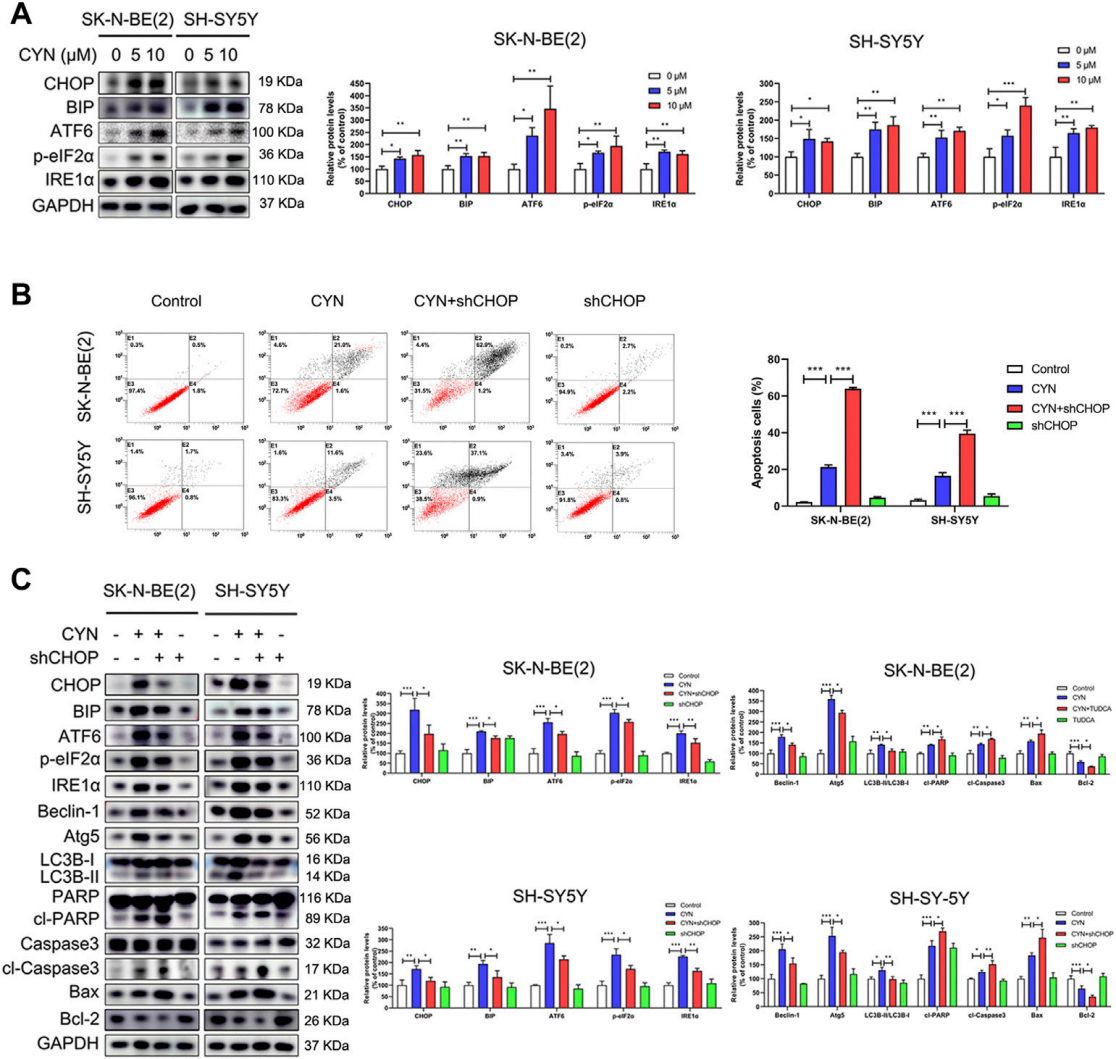
Relationship between ER stress, apoptosis and autophagy in NB cells treated by CYN. **(A)** Levels of ER stress-related proteins were analyzed by western blot. **(B)** Cell apoptotic ratio was measured and analyzed by flow cytometry. **(C)** Western blotting analysis detected the expression of ER stress-, autophagy- and apoptosis-related proteins. *p < 0.05, **p < 0.01, ***p < 0.001 versus the control group. *p < 0.05, **p < 0.01, ***p < 0.001 versus the CYN group.

Increasing evidence has indicated that ER stress is related to autophagy and apoptosis (Song et al., 2017). Considering the mutual connection among autophagy, apoptosis and ER stress, we explored the effects of CYN on ER stress components and its relationship with autophagy and apoptosis. First, we used western blotting to examine the expression levels of the ER stress markers CHOP, BIP, ATF6, p-eIF2α and IRE1α. As shown in Figure 5A, CYN (10 μM) treatment upregulated ER stress-associated proteins in a dose-dependent manner. Next, we ascertained the relationship among autophagy, apoptosis and ER stress in NB cells. CHOP is the downstream target for three branches of ER stress signalling, namely, eIF2α, ATF6 and IRE1α (Datta et al., 2019). Thus, shRNA targeting CHOP was used to inhibit ER stress, and autophagy and apoptosis were examined by flow cytometry and western blotting. The western blot results revealed that CHOP shRNA effectively reversed the expression levels of ER stress- and autophagy-associated proteins upregulated by CYN while further enhancing the expression levels of apoptotic proteins (Figure 5B). Moreover, the flow cytometry results showed that the apoptotic cell rate was increased in the CHOP shRNA and CYN cotreatment group compared with the CYN group (Figure 5C). To support the above results, we repeated the above experiments with the ER stress inhibitor TUDCA and obtained the same results (Supplementary Figure S3). Taken together, these results indicated that CYN triggered ER stress, which subsequently induced protective autophagy.

### CYN-induced autophagy inhibited apoptosis by promoting Nrf2 nuclear translocation and the P62-Keap1 interaction

**FIGURE 6:**
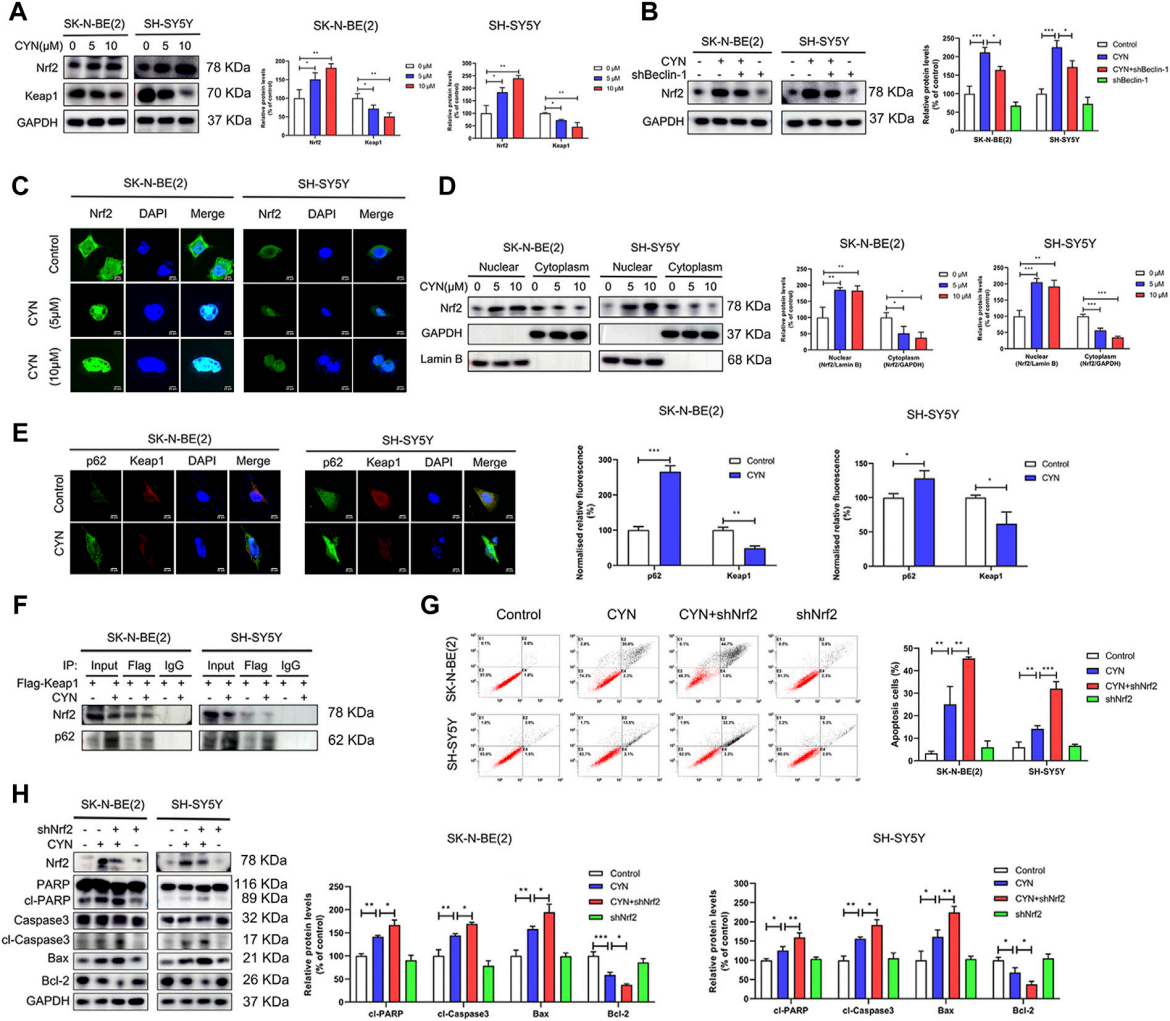
Relationship between the p62-Keap1-Nrf2 pathway and apoptosis in NB cells treated by CYN. **(A)** The Nrf2 and Keap1 protein level were determined by western blot. **(B)** Level of Nrf2 was analyzed by western blot. **(C)** CYN-treated NB cells were immunostained with anti-Nrf2 antibody (green) and counterstained with DAPI (blue). Scale bar: 20 µm. **(D)** The cytosolic and nuclear expressions of Nrf2 were determined by western blotting. **(E)** Immunofluorescence images and quantitative analysis of p62 and Keap1. Scale bars: 20 μm. **(F)** Nrf2 and p62 were detected by western blot after IP with anti-Flag. **(G)** Apoptosis of NB cells was measured and analyzed by flow cytometry. **(H)** Western blotting analysis detected the expression of apoptosis-related proteins. *p < 0.05, **p < 0.01, ***p < 0.001 versus the control group. *p < 0.05, **p < 0.01, ***p < 0.001 versus the CYN group.

Data from earlier studies have indicated that the Nrf2 pathway is involved in the antitumor activity of CYN in cancer cells (Takei et al., 2015; Ding et al., 2021). Given that p62 is an activator of the Nrf2-Keap1 pathway (Schmoll et al., 2017) and that the expression of p62 was upregulated by CYN stimulation (Figure 3D), we proposed that CYN could activate Nrf2 signalling in NB cells. Consistent with previous results (Takei et al., 2015; Ding et al., 2021), CYN (10 μM) treatment significantly increased Nrf2 expression in a dose-dependent manner (Figure 6A). The mRNA expressions of Nrf2 did not significantly change after CYN treatment (Supplementary Figure S4). Nrf2 plays a pivotal role in controlling redox balance, which attributed to the anti-cancer effects of drugs. Therefore, we detected the changes of reactive oxygen species (ROS) levels by flow cytometry to verify whether they are consistent with the trend of Nrf2 changes. As shown in Supplementary Figure S5, CYN induced the accumulation of ROS.To investigate whether there was an intrinsic link between autophagy and the Nrf2 pathway in CYN-treated NB cells, the protein level of Nrf2 was detected by western blotting. The results showed that treatment with shBeclin-1 reversed the increase in the expression of Nrf2 in CYN-treated NB cells (Figure 6B). Activation of Nrf2 signalling, indicated by Nrf2 nuclear translocation, has a critical role in autophagy. To illuminate whether CYN mediates the nuclear translocation of Nrf2, we observed the nuclear translocation of Nrf2 and monitored the subcellular localization of the Nrf2 protein by western blot analysis. Herein, we found that Nrf2 translocated from the cytosol to the nucleus after CYN treatment (Figure 6C), and the abundant accumulation of nuclear Nrf2 in CYN-treated NB cells was then confirmed by western blot analysis (Figure 6D). Under normal conditions, p62 competes with Keap1 for the same binding site on Nrf2; subsequently, Nrf2 is released from the Nrf2-Keap1 complex and translocates into the nucleus. As illustrated in Figure 6E, the immunofluorescence signal of p62 (green) was localized in the nucleus and cytoplasm, while the signal for Keap1 (red) was mainly distributed in the cytoplasm. Additionally, colocalization of p62 and Keap1 (yellow) was detected almost exclusively in the cytoplasm and the immunofluorescence intensity of p62 was enhanced, while that of Keap1 was weakened (Figure 6E). In addition, Co-IP showed that CYN stimulation increased the interaction between endogenous p62 and Keap1 but decreased the interaction between endogenous Nrf2 and Keap1 (Figure 6F). Next, we investigated the crosstalk between Nrf2 signalling and apoptosis, and the results showed that knockdown of Nrf2 significantly blocked the CYN-induced apoptosis (Figures 6G,H). Collectively, these data suggested that CYN-induced autophagy inhibited apoptosis through the p62/Keap1/Nrf2 pathways.

### CYN inhibited NB growth *in vivo*

**FIGURE 7:**
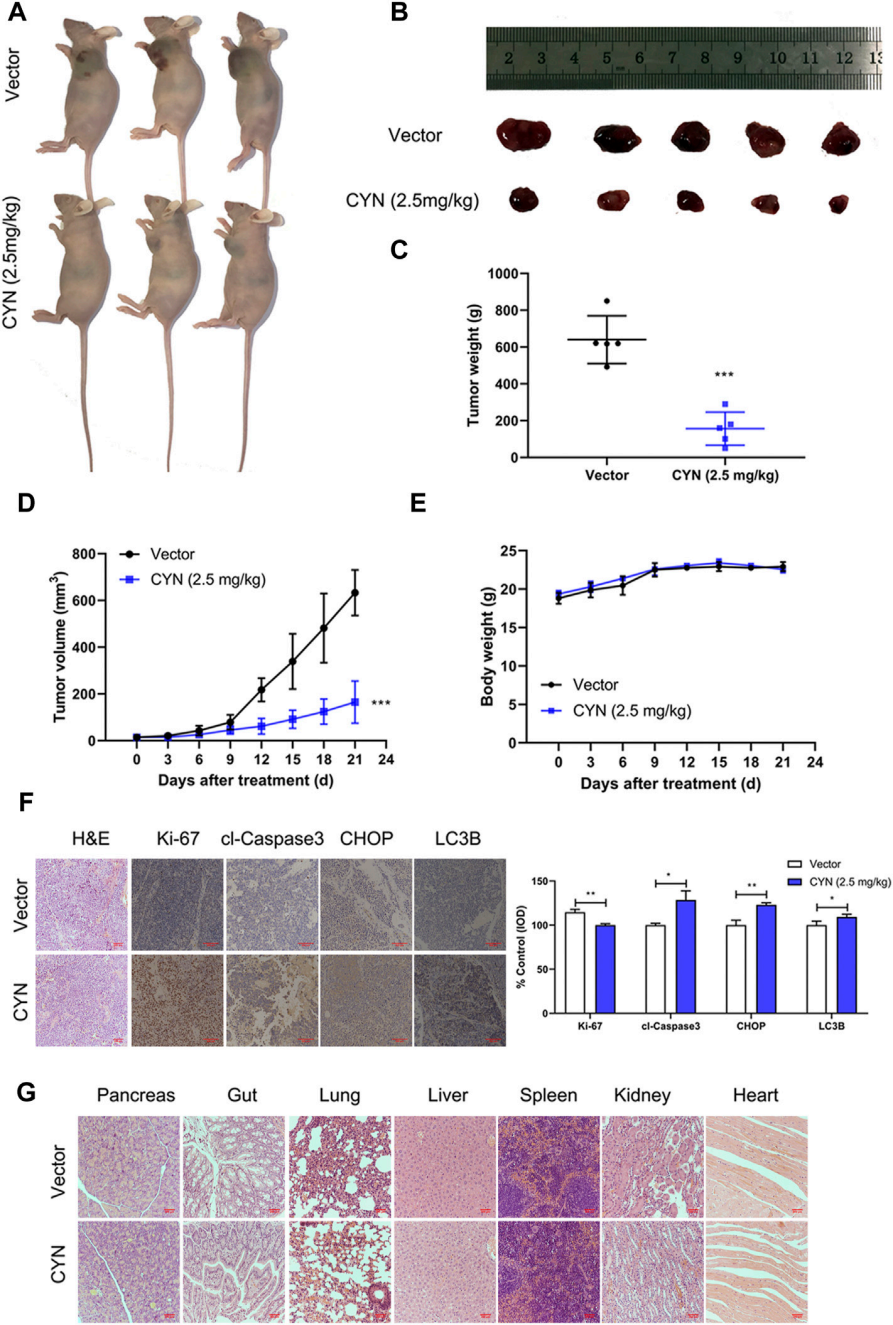
Effects of CYN on the NB tumor growth in athymic nude mice. **(A**,**B)** Representative images of xenograft tumors were shown. **(C)** Tumor weight after 3 weeks of treatment with CYN. **(D)** Tumor volume during administration of CYN. **(E)** The weight of mice was monitored during the experiment. **(F)** IHC staining results detected and analyzed the expression of Ki-67, cl-Caspase3, CHOP and LC3B. **(G)** HE stains examined the histopathologic characteristics of pancreas, gut, lung, liver, spleen, kidney and heart. *p < 0.05, **p < 0.01, ***p < 0.001 versus the vector group.

To investigate the antitumor properties of CYN *in vivo*, we subcutaneously inoculated SK-N-BE(2) cells into athymic nude mice, and after 1 week, the mice were intraperitoneally treated with 2.5 mg/kg CYN once a day for three consecutive weeks. Our results showed that CYN markedly decreased the tumour volumes and weights compared to the vehicle group (Figures 7A–D), with no effect on the mouse body weight (Figure 7E). Mouse tumour tissues were collected for IHC staining to confirm whether the results *in vivo* agreed with those *in vivo*. The data indicated that CYN treatment significantly upregulated the expression levels of cleaved caspase-3, CHOP and LC3B and downregulated the expression of Ki-67 compared with the vehicle group (Figure 7F). The H&E staining results revealed no notable pathological lesions in the pancreas, gut, lung, liver, spleen, kidney or heart tissues in the CYN treatment group (Figure 7G). Overall, these results demonstrated that CYN exerted potent antitumor properties with low toxicity in animals.

## Discussion

Currently, a large number of natural dietary compounds have attracted considerable attention because of their promising anticancer activity and good safety characteristics. CYN, a characteristic sesquiterpene lactone found in nutritious artichokes, has been shown to exhibit anticancer activity in cancer cell lines (Zheng et al., 2020; Ding et al., 2021). However, no previous study has reported the effect and underlying mechanisms of CYN in NB. In our study, we verified that CYN could significantly and concentration-dependently suppress the growth of NB SK-N-BE(2) and SH-SY5Y cells (Figure 1) with IC50 values of 9.731 μM in SK-N-BE(2) cells and 5.738 μM in SH-SY5Y cells (Figure 1D). Compared with other cancer cell lines, the IC50 values of CYN in NB cells were lower, suggesting that NB cells were much more sensitive to CYN than other cells (Ding et al., 2021). Our data showed that CYN suppressed the growth of NB cells by inducing G2 phase cell cycle arrest and apoptosis. Moreover, CYN treatment induced ER stress-dependent autophagy against apoptosis through the p62/Keap1/Nrf2 pathways.

CYN has been reported to induce apoptosis in various cancers (Zheng et al., 2020; Ding et al., 2021). Here, we found through flow cytometry analysis that, similar to the above results, CYN arrested the cell cycle at G2 phase, resulting NB cell apoptosis (Figure 2). Apoptosis, a type of programmed cell death, is regarded as a potential target for the antitumor actions of many anticancer drugs (Guicciardi and Gores, 2009). Moreover, apoptosis can be triggered in a caspase-dependent or a caspase-independent manner. In caspase-dependent apoptosis, apoptotic signalling occurs *via* two different pathways: the mitochondrial (endogenous) pathway and the death receptor (exogenous) pathway (Xiong et al., 2014). Notably, CYN appeared to induce apoptosis in NB cells through the mitochondrial apoptotic pathway, as evidenced by the activation of caspase-3 and PARP, the increased Bax protein level, and decreased Bcl-2 protein level (Figure 2C). Of course, it is worth noting that apoptosis is a type of RCD(regulated cell death), and it is extremely meaningful to add an omics part in future studies to systematically understand the molecular mechanism for studying the anticancer effect of CYN. Moreover, autophagy, as an evolutionarily conserved catabolic process, helps to maintain cellular homeostasis (Li et al., 2016). Given that autophagy is closely associated with apoptosis, we examined whether CYN affected autophagy in NB cells. We found that CYN significantly activated autophagy by increasing its initiation and blocking the fusion of autophagosomes and lysosomes (Figure 3). In a previous study, Cristiane et al. found that incubating bloodstream trypomastigotes with CYN induced autophagy (da Silva et al., 2013). However, there has been no report regarding the effect of CYN on autophagy in cancer, and further studies are needed. Autophagy is a highly dynamic multistep process, and both increasing the rate of autophagosome formation and decreasing the rate of autophagic degradation can promote the accumulation of autophagosomes. In this study, early-stage autophagy in CYN-treated NB cells was evidenced by an increased number of autophagosomes by TEM and increased protein expression of the autophagy markers LC3B-II, Atg5 and Beclin-1 (Figures 3A,B). On the other hand, with the aid of the mRFP-GFP-LC3B constructs, we demonstrated that CYN blocked autophagosome-lysosome fusion in these cells (Figure 3C). In addition, autophagic flux disruption by CYN through inhibition of autophagosome-lysosome fusion can be explained by the colocalization of LC3B with LAMP-2 (Supplementary Figure S1) and the p62 and LAMP-2 results (Figure 3D). Therefore, our study demonstrated that CYN promoted autophagy by initiating autophagy and inhibiting autophagic flux. ER stress is considered a common feature in several types of cancers (Bhardwaj et al., 2020; Xia et al., 2021).Additionally, previous evidence has suggested that various natural compounds and their derivatives can trigger ER stress to exert anticancer effects (Huang et al., 2021). Here, we showed for the first time that CYN activate ER stress in human cancer cells (Figure 5A). Based on the findings above, we were curious about the relationship among ER stress, apoptosis and autophagy after CYN treatment in NB cells.

There is a close relationship among ER stress, apoptosis and autophagy (Mizushima 2007; Cheng et al., 2015; Song et al., 2017). Our aim was to clarify the relationship among the three distinct processes, as it is essential during CYN treatment. First, we aimed to clarify the relationship between autophagy and apoptosis in CYN-treated NB cells. In recent years, increasing evidence has suggested that, inconsistent with apoptosis, autophagy plays contradictory roles in cancer and functions as a prosurvival or antisurvival mechanism depending on the tumour type or microenvironment (Healy et al., 2009; Song et al., 2017). In our study, we confirmed that inhibiting autophagy using siAtg5 and 3-MA (an early-phase autophagy inhibitor) increased apoptosis (Figures 4A,B). This observation suggests that autophagy plays a cytoprotective role in NB cells treated with CYN. In addition, we used chloroquine (CQ) to further investigate the relationship between autophagy and apoptosis. CQ, as a specific late-phase autophagy inhibitor, inhibits the fusion of lysosomes with autophagosomes (Boya et al., 2005). Our results demonstrated that the combination of CYN with CQ treatment reduced apoptosis compared with CYN treatment alone (Figures 4C,D). These results indicated that CYN-triggered blockade of autophagic lysosomal fusion played a crucial role in cytoprotective autophagy. Next, we aimed to clarify the relationship among ER stress, autophagy and apoptosis in CYN-treated NB cells. Accumulating evidence has suggested that ER stress plays a vital role in inducing apoptosis and autophagy in various tumour cells (Shen et al., 2017; Zheng et al., 2018). Moreover, ER stress can regulate autophagy alone or both autophagy and apoptosis (Shen et al., 2017; Zheng et al., 2018). In this study, the relationship among ER stress, apoptosis and autophagy was investigated after transfecting cells with CHOP-specific shRNA (Figure 5) or co-treated with ER stress inhibitor TUDCA (Supplementary Figure S3). Zheng et al. reported that the activation of ER stress could cause melanoma cell apoptosis and inhibit autophagy (Zheng et al., 2018). In contrast to previous studies, our results suggested that ER stress induced only protective autophagy in NB cells but did not participate in CYN-induced apoptosis. In line with our results, Shen et al. demonstrated that ER stress participated in only autophagy, not apoptosis, in sarcoma cells (Shen et al., 2017). These results indicated that CYN-triggered ER stress might induce protective autophagy in NB cells and then impair the antitumor effects of CYN. Therefore, it was necessary to further clarify the pro-autophagy mechanism of CYN treatment to improve the antitumour effects on NB.

In the present study, we observed that CYN increased the protein expression of p62 (Figure 3D). p62 acts as not only an autophagy receptor protein but also an activator of the noncanonical Keap1-Nrf2 pathway (Komatsu et al., 2010). It has also been documented that Nrf2 is activated in cancers, and the high activity of Nrf2 can play a protective role in tumour cells (Zhang et al., 2015; Rojo de la Vega et al., 2018). The interrelation between Nrf2 and p62 is well known; that is, p62 competitively binds with Keap1, which results in Nrf2 release from the Nrf2-Keap1 complex and Nrf2 translocation into the nucleus (Itoh et al., 2003; Schmoll et al., 2017; Dash et al., 2020). Our results showed that CYN improved Nrf2 transactivation by enhancing Nrf2 nuclear translocation, suggesting that CYN promoted Nrf2 activation by enhancing the binding of p62 to Keap1 in the cytoplasm (Figures 6C–F). It is noted that CYN enhanced the protein level of Nrf2 but not that of the mRNA. Thus, possibility exists that CYN may have enhanced Nrf2 protein expression *via* post-transcriptional mechanisms that deserve further study. Nrf2 signalling and autophagy crosstalk is complex and controversial. The study conducted by Xu et al. revealed that Nrf2 acted as a downstream regulator of autophagy in gastric cancer cells (Xu et al., 2022). In contrast, other findings from the study by Xu et al. reported a negative interaction between autophagy and the Nrf2 pathway (Li et al., 2018). Our findings were in agreement with Xu et al.’s results (Li et al., 2018), and the meaningful downregulation of Nrf2 protein expression following the inhibition of autophagy induced by shBeclin-1 (Figure 6B) suggested that Nrf2 signalling could be partly activated by autophagy in CYN-treated NB cells. The association between autophagy, apoptosis and NRF2 pathway has been gradually concerned and studied in recent years (Arab et al., 2021; Lu et al., 2021). To verify that CYN-induced autophagy inhibits apoptosis through the Nrf2 pathway, we investigated the crosstalk between the two signalling pathways. Our findings were in agreement with Xu et al.’s results (Xu et al., 2022), in which Nrf2 knockdown inhibited apoptosis in CYN-treated NB cells (Figures 6G,H), indicating that Nrf2 signalling might be a disadvantageous element for the antitumor effects of CYN on NB. Furthermore, we preliminarily detected the changes of ROS after CYN treatment (Supplementary Figure S5), suggesting that there may be an exploratory association between antioxidant response and autophagy in neuroblastoma treated with CYN, which is worthy of further study in the future.

**FIGURE 8:**
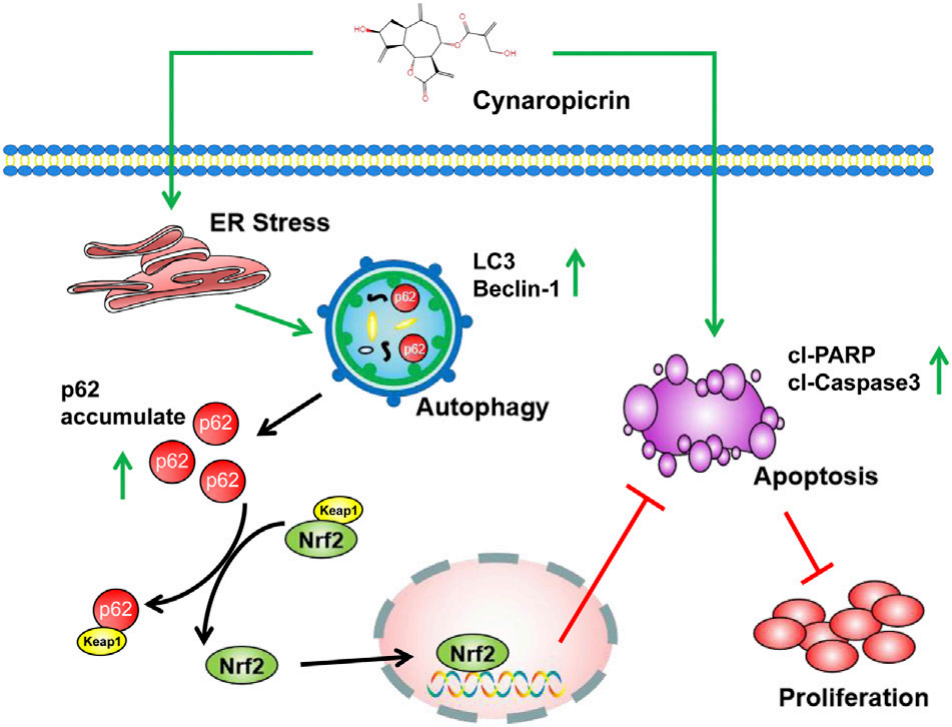
Schematic diagram of the anti-NB effects of CYN.

In summary, the current study indicated that CYN inhibited NB cell growth *in vitro* and *in vivo*, and the mechanism specifically involved ER stress/autophagy/Nrf2 signalling/apoptosis. We provided insights into the molecular mechanisms by which CYN induces apoptosis and ER stress-mediated protective autophagy. That is, ER stress-mediated autophagy triggers the p62/Keap1/Nrf2 pathways, followed by attenuation of CYN-induced apoptosis in NB; the interplay is summarized in Figure 8. Our study indicated that CYN may be a potential antitumor agent for NB prevention and treatment. This is the first study to examine the crosstalk between CYN-induced apoptosis and autophagy, which involves the activation of the p62/Keap1/Nrf2 signalling pathway. Therefore, we provided a basis for future preclinical and clinical trial exploration to improve the efficacy of CYN in NB treatment.

## Data Availability

The original contributions presented in the study are included in the article/Supplementary Material, further inquiries can be directed to the corresponding authors.
