# Supplementary figures and images for: Suppression of endoplasmic reticulum stress-dependent autophagy enhances cynaropicrin-induced apoptosis via attenuation of the P62/Keap1/Nrf2 pathways in neuroblastoma
Source: Front Pharmacol. 2022 Sep 16;13:977622. doi: 10.3389/fphar.2022.977622 (PMC9523313; doi:10.3389/fphar.2022.977622)

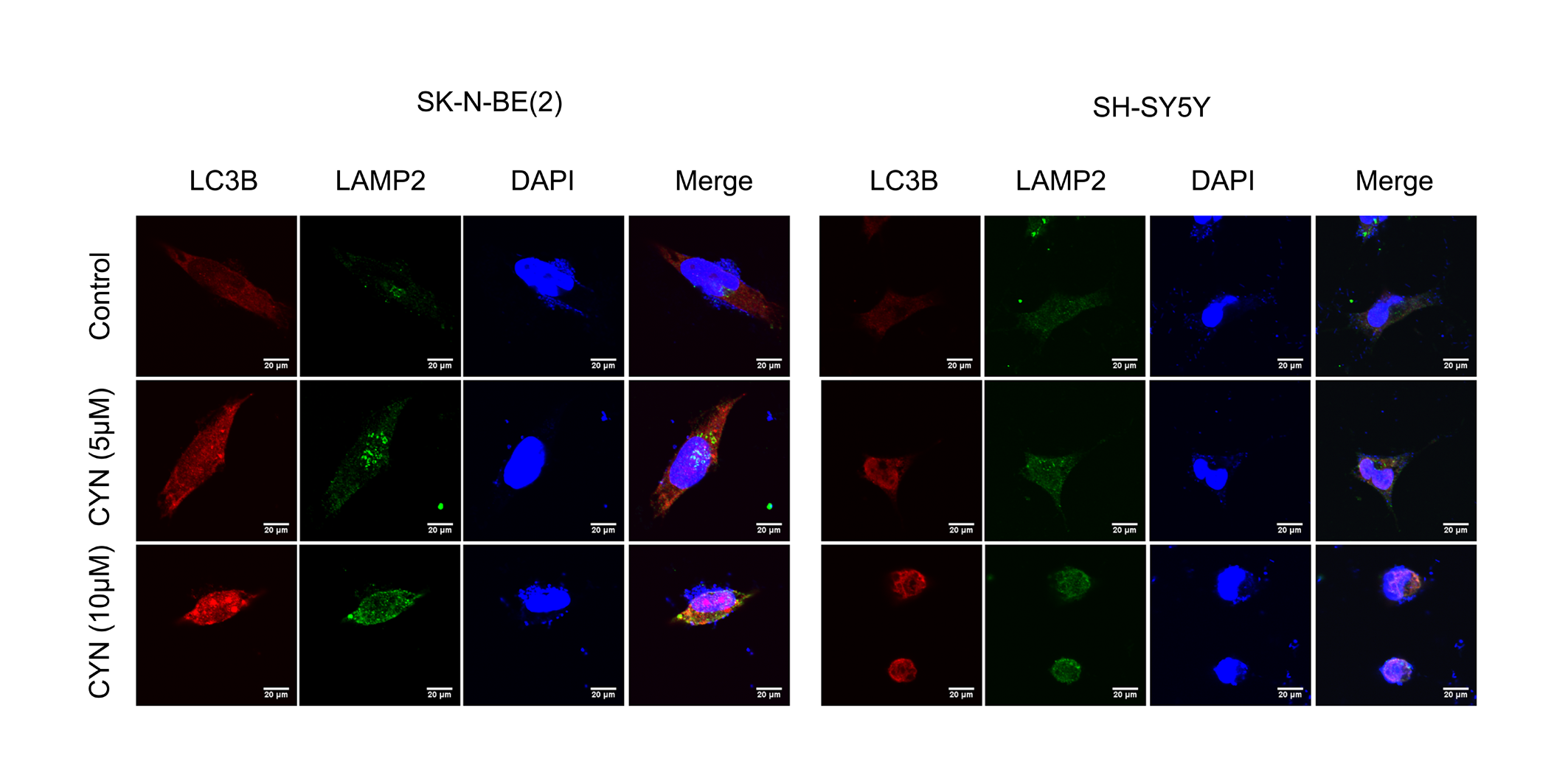

Supplement: Supplementary file 1 [file DataSheet1.zip › Supplementary figures/Figure S1.tif]

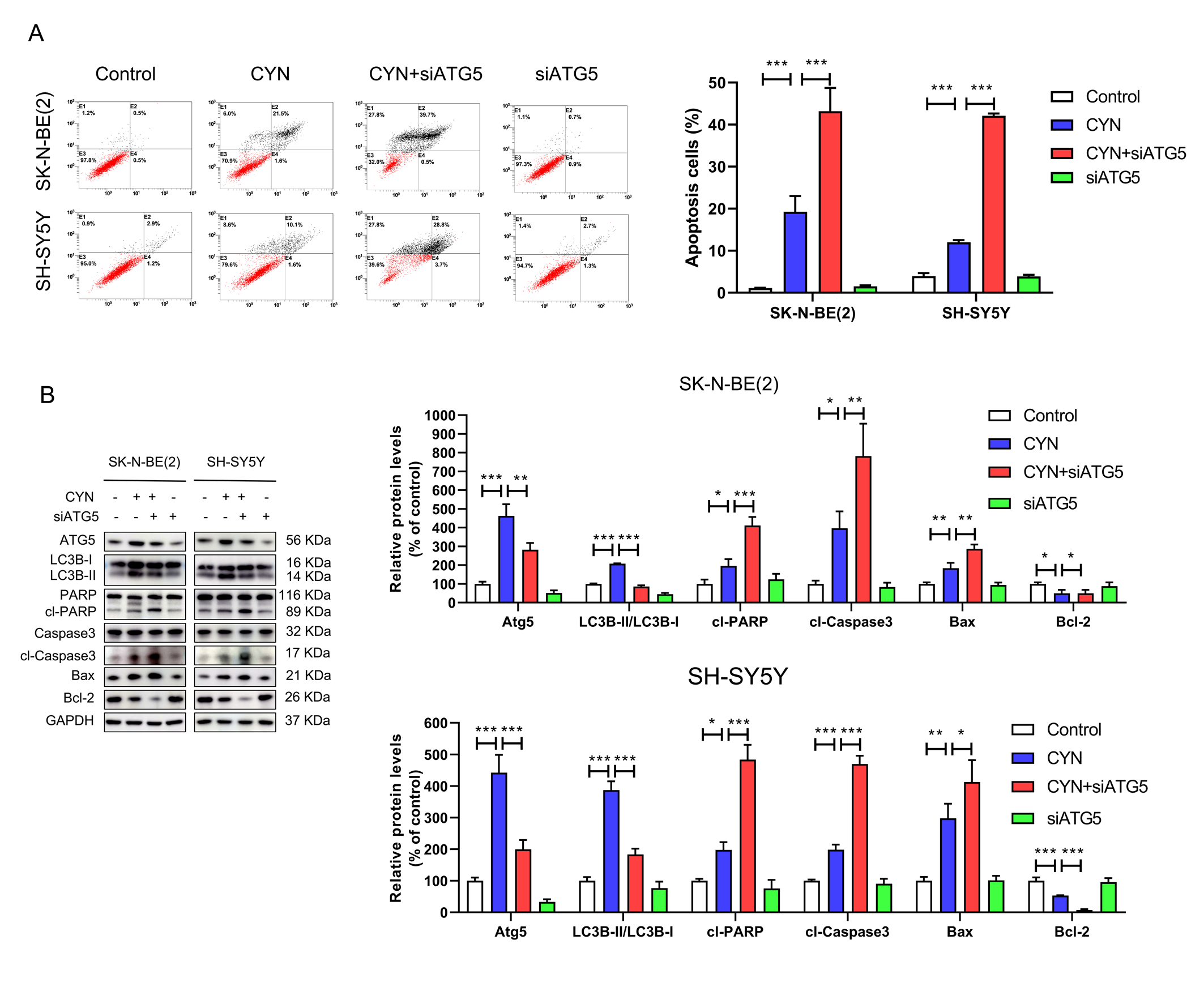

Supplement: Supplementary file 1 [file DataSheet1.zip › Supplementary figures/Figure S2.TIF]

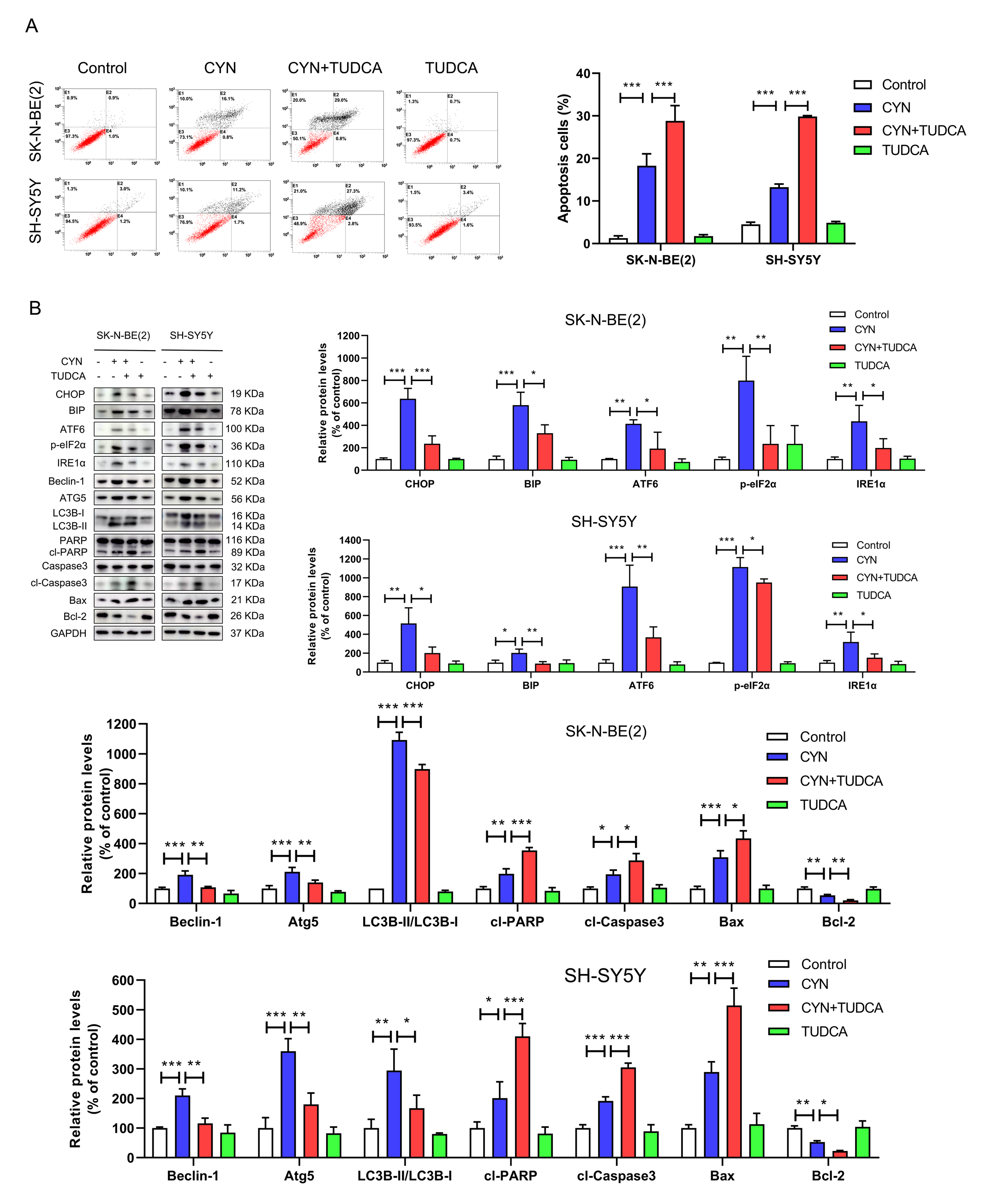

Supplement: Supplementary file 1 [file DataSheet1.zip › Supplementary figures/Figure S3.TIF]

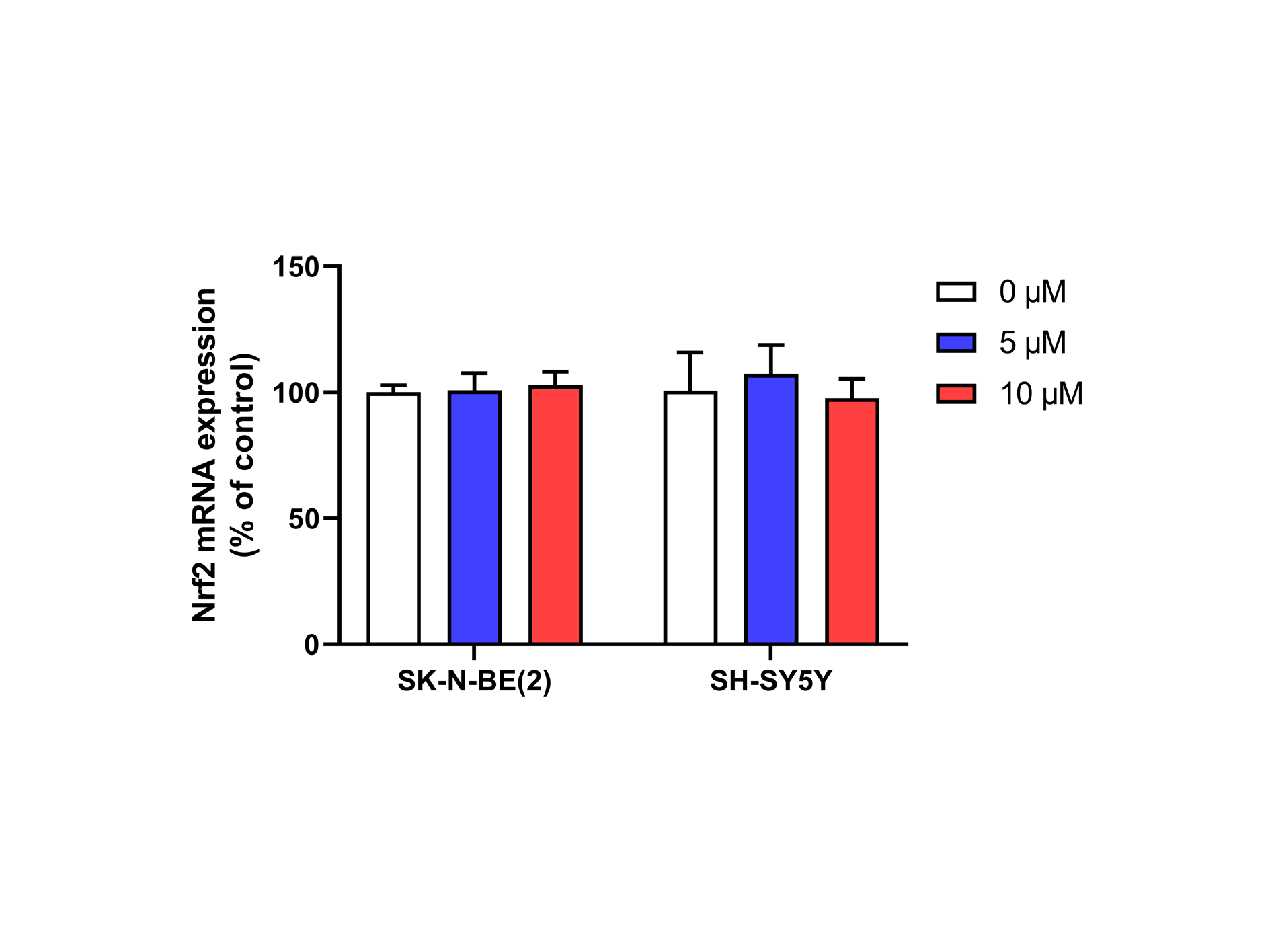

Supplement: Supplementary file 1 [file DataSheet1.zip › Supplementary figures/Figure S4.tif]

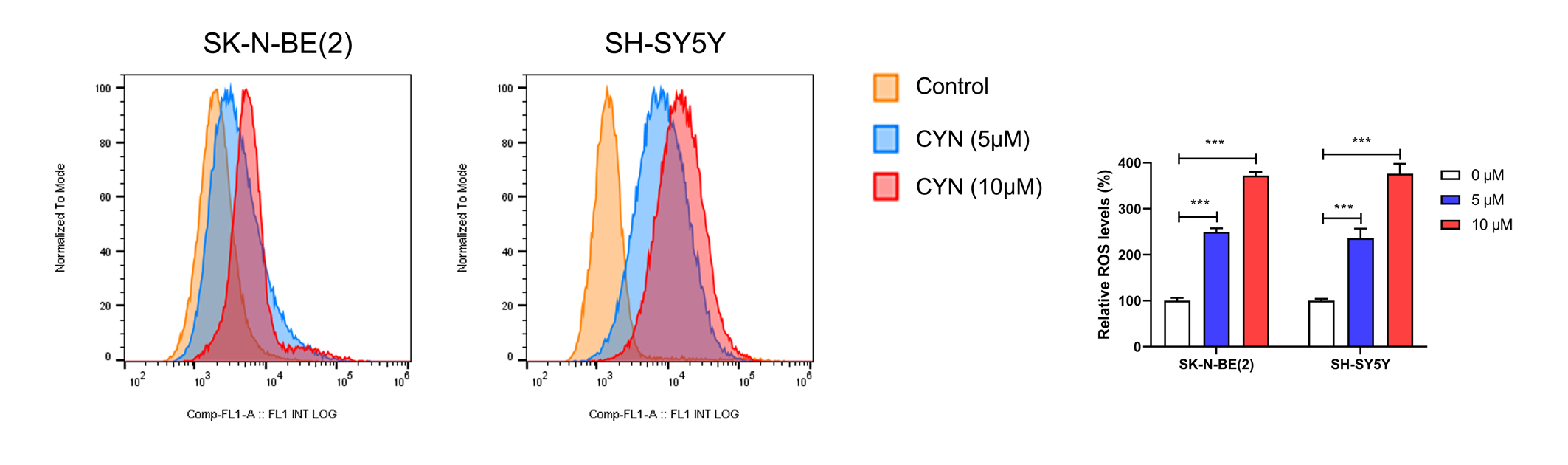

Supplement: Supplementary file 1 [file DataSheet1.zip › Supplementary figures/Figure S5.tif]
