# Supplementary material for: Suppression of endoplasmic reticulum stress-dependent autophagy enhances cynaropicrin-induced apoptosis via attenuation of the P62/Keap1/Nrf2 pathways in neuroblastoma
Source: Front Pharmacol. 2022 Sep 16;13:977622. doi: 10.3389/fphar.2022.977622 (PMC9523313; doi:10.3389/fphar.2022.977622)
Supplement: Supplementary file 1 [file DataSheet1.zip › Supplementary figures/Supplementary figure legends.doc]

**Supplementary figure legends**

**FIGURE S1** The colocalization of LAMP2 and LC3B. The fluorescence of LAMP2 and LC3B was detected by confocal microscopy to determine their colocalization in NB cells.

**FIGURE S2** SiATG5-mediated inhibition of autophagy effectively increased CYN-induced apoptosis in NB cells.

**FIGURE S3** TUDCA-induced inhibition of ER stress effectively inhibited CYN-induced autophagy, thereby increasing apoptosis in NB cells. **(A)** The number of apoptotic cells was assessed by Annexin V-FITC/PI staining and flow cytometry. **(B)** The expression levels of autophagy and apoptosis markers were measured by western blot analysis. *P < 0.05, **P < 0.01, ***P < 0.001 versus the control group. *P < 0.05, **P < 0.01, ***P < 0.001 versus the CYN group.

**FIGURE S4** CYN had no effect on Nrf2 mRNA levels. The mRNA expressions of Nrf2 in NB cells was calculated against the housekeeping gene GAPDH.

**FIGURE S5** CYN induced the accumulation of reactive oxygen species (ROS). **(A)** The number of apoptotic cells was assessed by Annexin V-FITC/PI staining and flow cytometry. **(B)** The expression levels of autophagy and apoptosis markers were measured by western blot analysis. *P < 0.05, **P < 0.01, ***P < 0.001 versus the control group. *P < 0.05, **P < 0.01, ***P < 0.001 versus the CYN group.
